# Persistence of type-specific human papillomavirus infection among Daqing City women in China with normal cytology: a pilot prospective study

**DOI:** 10.18632/oncotarget.20188

**Published:** 2017-08-11

**Authors:** Ni Li, Dong Hang, Lin Yang, Xiaoshuang Feng, Zhangyan Lyu, Shuanghua Xie, Jing Zhou, Lingying Wu, Xiaoguang Li, Nan Li, Min Cheng, Kai Zhang, Zhihui Zhang, Hong Cui, Jian Yin, Zhibin Hu, Hongbing Shen, Min Dai

**Affiliations:** ^1^ Program Office for Cancer Screening in Urban China, National Cancer Center/Cancer Hospital, Chinese Academy of Medical Sciences and Peking Union Medical College, Beijing, China; ^2^ Department of Epidemiology and Biostatistics, Collaborative Innovation Center of Cancer Medicine, School of Public Health, Nanjing Medical University, Nanjing, China; ^3^ Department of Hospital Infection Control, Beijing Jishuitan Hospital, Fourth Medical College of Peking University, Beijing, China; ^4^ Department of Gynecology Oncology, National Cancer Center/Cancer Hospital, Chinese Academy of Medical Sciences and Peking Union Medical College, Beijing, China; ^5^ Department of Cancer Prevention, National Cancer Center/Cancer Hospital, Chinese Academy of Medical Sciences and Peking Union Medical College, Beijing, China; ^6^ Department of Pathology, National Cancer Center/Cancer Hospital, Chinese Academy of Medical Sciences and Peking Union Medical College, Beijing, China

**Keywords:** human papillomavirus, type-specific persistence, cervical cancer, prospective study

## Abstract

Persistence of high-risk human papillomavirus (HPV) represents the necessary cause of cervical cancer. Researching natural history of HPV infection is important to identify high-risk population of cervical cancer. Since HPV infection is population-specific, the findings in western populations could not be simply extended to Chinese and Asian females. This study investigated the type-specific persistence of HPV and related factors among Daqing City women in China. A total of 1759 women aged 18–80 years were enrolled at baseline. Cervical cell specimens were collected for cytological examination and HPV detection. HPV-positive individuals with normal cytology were followed up after 12 months. The results showed that HPV prevalence was 8.64% at baseline, of which 85 HPV-positive cases with normal cytology were followed up. The one-year type-specific persistence of HPV and high-risk types were 34.12% (29/85) and 34.25% (25/73), respectively. The top three high-risk types were HPV16 (7/17, 41.18%), HPV18 (5/8, 62.50%) and HPV58 (7/15, 46.67%). Age > 50 years was significantly associated with a higher risk of HPV persistence compared to ≤ 50 (OR = 2.73; 95% CI: 1.07, 6.93). In conclusion, approximately one-third of Daqing City women with HPV infection had at least one-year viral persistence, most of which were high-risk types. Older age represents a risk factor of HPV persistence.

## INTRODUCTION

With wide acceptance of human papillomavirus (HPV) as a necessary cause of cervical cancer [1], primary and secondary cervical cancer prevention strategies have moved towards detection and control of the virus. However, although approximately 80% of women will acquire an HPV infection during their life time [2], the majority of infections will be cleared and only a very small proportion can lead to cervical intraepithelial neoplasia (CIN) and cervical cancer. Persistent infection with high-risk oncogenic HPV, rather than transient infection, has been found to be the genuine factor associated with the development of cervical cancer [3]. Hence, although HPV DNA test is widely used as an adjuvant method of cytology examination for the identification of high-risk population of cervical cancer, many transient infections without oncogenic significance decrease the specificity of HPV DNA test. It is necessary to make a better understanding of HPV persistence for cost-effectiveness of HPV test in cervical cancer screening programs.

Several studies on persistent infection of HPV have been carried out in women from different countries, and revealed the persistence of HPV infection varied between 2.4% to 54% with 12–60 months [4–10]. The large variation of the persistence estimates might be due to differences in definitions of persistence, the interval and length of follow-up, HPV DNA detection methods, and study populations. According to a published meta-analysis [11], most studies defined persistence as HPV positivity at two or more time points, whereas others used three or more HPV-positive visits, the proportion of HPV-positive visits, HPV positivity throughout follow-up, or time to clearance. Most studies included women with normal cytology at baseline, with the PCR and hybrid capture assay being the most commonly used methods to detect HPV. HPV16 is the most frequent type worldwide, whereas the distribution of other types varies geographically. For example, HPV18 is the second leading type in Europe and South America, while HPV52 and HPV58 are more dominant in Asia [12]. HPV16, 31, 33 and 52 were shown to be more persistent than other types [11].

HPV vaccination has not been administrated in China, and characterizing type-specific HPV persistence in the target population will benefit the evaluation of HPV vaccination effect in the future. Thus, we performed a prospective study to determine the type-specific HPV persistence, as well as potential risk factors in Daqing City women. To the best of our knowledge, this is the first population-based study on type-specific HPV persistence among Chinese women.

## RESULTS

In the follow-up of 152 HPV-positive cases identified from 1759 participants at baseline, 67 (44.08%) cases refused to participate after 12 months. But no significant difference in primary socio-demographics was found between those attending the follow-up and those refusing (all *P* > 0.05, Table 1).

**Table 1:** Social-demographic characteristics between HPV-positive women who attended and who refused the follow-up

Variable	Follow-up (*n* = 85) *N* (%)	Lost (*n* = 67) *N* (%)	P*
Age (year)			0.805
20–34	12 (14.12)	13 (19.40)	
35–44	37 (43.53)	23 (34.33)	
45–54	24 (28.24)	20 (29.85)	
55–64	9 (10.59)	8 (11.94)	
65–79	3 (3.53)	3 (4.48)	
Ethnicity			0.385
Han	80 (95.24)	65 (98.48)	
Others	4 (4.76)	1 (1.52)	
Education			0.327
High school and below	34 (40.48)	32 (48.48)	
College and above	50 (59.52)	34 (51.52)	
Marital status			0.959
Single, widowed, or divorced	15 (17.86)	12 (18.18)	
Married	69 (82.14)	54 (81.82)	
Family monthly income (Chinese yuan)			0.422
< 5000	39 (46.43)	35 (53.03)	
≥ 5000	45 (53.57)	31 (46.97)	
Cigarette smoking			0.385
No	80 (95.24)	65 (98.48)	
Yes	4 (4.76)	1 (1.52)	

HPV, human papillomavirus.

*The χ^2^ test was used.

At baseline, the most prevalent types of HPV infection included HPV16 (1.88%), HPV52 (1.65%), HPV58 (1.08%), and HPV18 (0.91%) (Table 2). Excluding HPV types of very low prevalence (< 0.5%), we found that HPV18 (62.50%), HPV58 (46.67%), and HPV16 (41.18%) were the most common high-risk types of persistence.

**Table 2:** Cervical type-specific persistence of HPV among women in Daqing City, China

HPV type	Baseline (*n* = 1759)		Follow-up (*n* = 85)	
	*N*	Prevalence (%)	*N*	Persistence (%)
Any type	152	8.64	29/85	34.12
High-risk	131	7.45	25/73	34.25
HPV16	33	1.88	7/17	41.18
HPV18	16	0.91	5/8	62.50
HPV26	3	0.17	0/0	-
HPV31	5	0.28	0/0	-
HPV33	10	0.57	1/4	25.00
HPV39	15	0.85	1/7	14.29
HPV45	2	0.11	0/2	0.00
HPV51	3	0.17	1/2	50.00
HPV52	29	1.65	2/13	15.38
HPV56	2	0.11	1/1	100.00
HPV58	19	1.08	7/15	46.67
HPV59	2	0.11	2/2	100.00
HPV66	1	0.06	0/1	0.00
HPV68	7	0.40	0/4	0.00
Low-risk	29	1.65	4/16	25.00
HPV6	9	0.51	0/6	0.00
HPV11	1	0.06	0/0	-
HPV40	1	0.06	0/0	-
HPV53	1	0.06	0/1	0.00
HPV55	2	0.11	0/0	-
HPV61	13	0.74	4/8	50.00
HPV82	4	0.23	0/1	0.00

HPV, human papillomavirus.

The results of risk factors associated with HPV persistence were presented in Table 3. Age > 50 years was significantly associated with a higher risk of HPV persistence compared to ≤ 50 (OR = 2.73; 95% CI: 1.07, 6.93). However, we did not find significant associations of persistent infection with the other lifestyle factors.

**Table 3:** Risk factors associated with type-specific HPV persistence

Variable	No. of HPV persistence	No. of HPV transient infection	OR (95% CI)	*P*
Age (year)				
≤ 50	10	33	1.00	0.033
> 50	19	23	2.73 (1.07, 6.93)	
Education				
High school and below	11	23	1.00	0.730
College and above	18	32	1.18 (0.47, 2.96)	
Marital status				
Single, widowed, or divorced	7	8	1.00	0.275
Married	22	47	0.53 (0.17, 1.66)	
Family monthly income (yuan)				
< 5000	14	25	1.00	0.805
≥ 5000	15	30	0.89 (0.36, 2.20)	
Cigarette Smoking				
No	27	53	1.00	0.505
Yes	2	2	1.96 (0.26, 14.71)	
Alcohol Drinking				
No	26	53	1.00	0.217
Yes	3	2	3.06 (0.48, 19.44)	
Family history of cancer				
No	18	28	1.00	0.246
Yes	10	27	0.58 (0.23, 1.47)	
Age of menarche (year)				
≤ 15	19	30	1.00	0.332
> 15	10	25	0.63 (0.25, 1.60)	
Regularmenstrual cycle				
Yes	26	50	1.00	0.852
No	3	5	1.15 (0.26, 5.21)	
Age of first sexual behavior(year)				
≤ 24	16	36	1.00	0.356
> 24	13	19	1.54 (0.61, 3.86)	
Number of sexual partners				
1	27	48	1.00	0.411
≥ 2	2	7	0.51 (0.10, 2.62)	
Condom use				
Ever	6	9	1.00	0.483
Never	18	41	0.66 (0.20, 2.13)	
Oral contraceptiveuse				
Ever	1	1	1.00	0.591
Never	23	49	0.47 (0.03, 7.84)	
HPV at baseline				
Single	23	52	1.00	0.066
Multiple	6	4	3.39 (0.87, 13.17)	

HPV, human papillomavirus; OR, odds ratio; CI, confidence interval.

## DISCUSSION

In this prospective cohort, 8.6% of women aged 18–80 years had cervical infections of HPV at baseline survey. Follow-up data from HPV-positive women enabled us to estimate HPV persistence (34.1%), with 34.3% persistence of high-risk types. We found that older age (> 50 years) was a risk factor for persistent infection. To our best knowledge, this study provided the first evidence on population-based type-specific HPV persistence in Chinese women.

Pooled analysis of the International Agency for Research on Cancer (IARC) HPV prevalence surveys showed variation of nearly 20 times (1.4%–25.6%) in age-standardized HPV prevalence between different areas [13]. The reported prevalence in Asia was 8.7% (95% CI: 7.9%–9.5%) [13], similar to the data in our study (8.6%). It was suggested that the persistence of HPV infection was approximately 30–50% with 12-months follow-up [7, 9, 10, 14]. In our prospective study, a similar persistence (34.1%) was estimated among Daqing City women with normal cytology. HPV16 and HPV18 represent the most common oncogenic types worldwide, while HPV58 is more frequently detected in East Asia [15]. We also confirmed that HPV16, 18 and 58 infections were more likely to persist in our cohort.

Previous studies have suggested that both viral and host factors are implicated in persistent HPV infection [16]. In the aspect of virus, viral load, multiple infections, and certain types or variants were reported to influence HPV persistence [17–19]. Meanwhile, host factors such as number of sexual partners, condom use, oral contraceptive, and immunodeficiency have also been associated with the risk of HPV persistence [10, 20, 21]. In our study, we only found a significant association between age and persistent HPV infection, which was consistent with several previous studies [5, 7, 22]. One possible explanation for this association is that immune function is gradually weakened during aging, which may result in HPV evasion from host immune system [23]. Besides, prevalent infections in older women are also likely to represent those that have already persisted a long time. Because the numbers of women reporting to have high number of sexual partners, condom use, and oral contraceptives were all small in this study, other life-style related factors are needed to be identified. We also note that the associations with life-style related factors for HPV persistence remained insignificant after adjustment of the models for age.

The limitation of this study is that some persistent infections might be a re-infection with the same type due to the interval of approximately 12 months between baseline and follow-up. A shorter interval of HPV detection could improve the estimation of HPV persistence. However, repeat HPV testing at 12 months intervals is widely used in cervical screening programs to identify women at high risk of cervical precancerous lesion [24]. In addition, the sample size of our study is relatively small, which may limit the statistical power to identify risk factors. Strengths of our population-based study include the use of standardized in-person interviews conducted by trained interviewers in a private one-on-one setting that included detailed questions on sensitive risk behaviors and inclusion of only cytologically confirmed women with normal cervix in our study, which minimized the possibility of selection bias.

In conclusion, this pilot prospective study investigated the type-specific persistence of HPV among Daqing City women with normal cytology. Further large-scale and long-term follow-up studies are warranted to improve targeted screening and effective prevention.

## MATERIALS AND METHODS

### Study population and follow-up

This prospective study was conducted in a community of the Sartu District, Daqing City, China. All mentally and physically competent women aged 18–80 years were invited to Daqing Aixin Hospital to participate in the baseline survey in 2010. After exclusion of 55 women refusing to participate, and 201 unmarried, pregnant or hysterectomized women who did not undergo gynecological examination and provide cervical cell specimens, a total of 1759 women were enrolled at baseline. According to the Bethesda 2001 nomenclature, a diagnosis by liquid-based cytology was assigned to each participant as having negative for intraepithelial lesion or malignancy (NILM) or having an epithelial cell abnormality such as atypical squamous cells of undetermined significance (ASCUS), low-grade squamous intraepithelial lesion (LSIL), high-grade squamous intra epithelial lesion (HSIL) and invasive cervical cancer (ICC). HPV DNA was detected in 152 (8.64%) women with normal cytology (NILM). Of them, 85 (55.92%) women participated in the follow-up after 12 months. This study was approved by the ethics committee of Cancer institute and Hospital, Chinese Academy of Medical Sciences (CICAMS). All participants signed an informed consent form according to the recommendations of CICAMS.

### Socio-demographic and behavioral data

All enrolled women were interviewed by well-trained nurses in a separate room in Daqing Aixin Hospital. The structured questionnaires included information on socio-demographic characteristics, reproductive and menstrual factors, sexual habits, and contraceptive methods.

### Cervical specimen collection and HPV DNA detection

Gynecological examination and cervical cell collection were described in our previous studies [25]. TellgenplexTM HPV DNA Test (Tellgen Life Science, Shanghai, China), a polymerase chain reaction (PCR)-based fluorescent Luminex assay [26] was applied to detect 24 HPV types including 15 high-risk types (HPV16, 18, 26, 31, 33, 35, 39, 45, 51, 52, 56, 58, 59, 66, 68) and 9 low-risk types (HPV6, 11, 40, 42, 44, 53, 55, 61, 82). Briefly, HPV DNA was amplified by multiplex PCR using biotin-labeled consensus PCR primers. The PCR products were then hybridized to sets of color-coded microspheres. Each set of microspheres was coated with a pre-designed HPV type-specific probe. After incubation with phycoerythrin-conjugated streptavidin, microspheres were read by using a Luminex 200 system (Luminex Corporation, TX, USA). HPV types were determined according to the unique fluorescent dye signature from each set of microspheres, when the signature value was > 150.

### Statistical analysis

The χ^2^ test was used to assess soci-demographic differences between individuals attending and refusing the follow-up. Odds ratios (ORs) for HPV persistence and corresponding 95% confidence intervals (CIs) were calculated by logistic regression equations. All tests were two-sided and *P* < 0.05 was defined as statistically significant.

## Abbreviations

HPV: human papillomavirus
CIN: cervical intraepithelial neoplasia
NILM: negative for intraepithelial lesion or malignancy
ASCUS: atypical squamous cells of undetermined significance
LSIL: low-grade squamous intraepithelial lesion
HSIL: high-grade squamous intra epithelial lesion
ICC: invasive cervical cancer
OR: odds ratios
CI: confidence interval
IARC: International Agency for Research on Cancer.
